# The complete chloroplast genome sequence of coastal psammophyte*, Ixeris repens* (Asteraceae, subtribe Crepidinae), in Korea

**DOI:** 10.1080/23802359.2021.1899076

**Published:** 2021-03-19

**Authors:** Woong Lee, JiYoung Yang, Seung-Chul Kim, Jae-Hong Pak

**Affiliations:** a Research Institute for Dok-do and Ulleung-do Island, Kyungpook National University, Daegu, Republic of Korea; b Department of Biological Sciences, Sungkyunkwan University, Suwon, Republic of Korea; c Research Institute for Dok-do and Ulleung-do Island, Daegu, Republic of Korea; d Department of Biology, School of Life Sciences, Kyungpook National University, Daegu, Republic of Korea

**Keywords:** Subtribe Crepidinae, *Ixeris repens*, psammophyte, plastid genome

## Abstract

We report the first complete chloroplast genome sequence of psammophyte, *Ixeris repens*, on the coastal dunes in Korea. The complete plastid genome is 153,017 bp in total length, with one large single copy (LSC; 84,242 bp), one small single copy (SSC; 18,495 bp), and two inverted repeat (IR) regions (IR_a_ and IR_b_, each with 25,140 bp). The overall GC content is 37.6% and the genome contains 130 genes, including 85 protein-coding, 37 transfer RNA and 8 ribosomal RNA genes. Phylogenetic analysis based on 17 representative plastomes of the family Asteraceae suggests that *Ixeris repens* is sister to congeneric species *I. polycephala* with strong bootstrap support (100%) and also that monophyletic *Ixeris* is sister to the clade containing *Taraxacum*, *Youngia*, *Lapsanastrum*, and *Crepidiastrum*.

The subtribe Crepidinae as defined by Kilian et al. (2009) is the largest subtribe of the tribe Cichorieae (also known as Lactuceae Cass.). A preliminary molecular phylogeny of Cichorieae based on nuclear ribosomal DNA (nrDNA) internal transcribed spacer (ITS) region has identified five major clades within the subtribe Crepidinae: *Crepis*-*Lapsana*-*Rhagadiolus* clade, *Crepidiastrum*-*Lapsanastrum*-*Youngia* clade, *Ixeris*-*Ixeridium*-*Taraxacum* clade, *Garhadiolus*-*Heteracia* clade, and *Dubyaea*-*Nabalus*-*Soroseris*-*Syncalathium* clade (Kilian et al. 2009). In the *Ixeris*-*Ixeridium*-*Taraxacum* clade, the two genera, *Ixeris* and *Ixeridium*, are in sister group relationship and *Taraxacum* appears to be closely related to this clade (Enke and Gemeinholzer 2008; Nakamura et al. 2014).

The genus *Ixeris* comprises close to ten species and is centered in East Asia, extending to South and Southeast Asia. Based on carpological and cytological investigations, a taxonomic revision of *Ixeris* and its relatives was provided by Pak and Kawano (1990a, b, c, 1992), which is largely in agreement with the narrow generic concept of Nakai (1920). Of six *Ixeris* species in Korea, *I. repens* (L.) A.Gray shows very wide geographic distribution, ranging from Indochina (Cambodia, Thailand, Vietnam, and Laos) through Taiwan, China, Japan, Korea to the Russia Far East (Kamchatka). *Ixeris repens* can be found commonly on the coastal dunes of western and southeastern parts of the Korean Peninsula as well as on Jeju-do Island. As one of vulnerable coastal species, nearly 18% of reduction in the potential distribution area of *I. repens* and future potential distribution shift toward the northern parts along the east coast in the Korean Peninsula have been suggested (Park and Choi 2020). Although phylogenetic relationships within *Ixeris* haven not been fully determined, nrDNA ITS sequence data showed that *I. repens* is closely related to *I. stolonifera* A.Gray, which occurs in open mountain slopes and disturbed areas (Nakamura et al. 2014). In this study, we assembled the first complete plastome of *I. repens*, analyzed available plastomes of *Ixeris* and related genera, and assessed its phylogenetic position and intergeneric relationships within the subtribe Crepidinae.

Total DNA (Voucher specimen geographic coordinates: 35°22′40″N, 129°20′40″E) was isolated using the DNeasy plant Mini Kit (Qiagen, Carlsbad, CA) and sequenced by the Illumina platform (Macrogen, Seoul, Korea). The specimen was deposited at Kyungpook National University Herbarium (KNU; http://bio.knu.ac.kr/PhD/profile/lst.do?seq=6, Jae-Hong Pak, jhpak@knu.ac.kr) under the voucher specimen of “Lee200630019”. A total of 28,694,152 paired-end reads were obtained and assembled *de novo* with Velvet v. 1.2.10 using multiple *k*-mers (Zerbino and Birney 2008). The tRNAs were confirmed using tRNAsacn-SE (Lowe and Eddy 1997). The complete plastome length of *I. repens* (MW092111) was 153,017 bp, with large single copy region (LSC; 84,242 bp), small single copy region (SSC; 18,495 bp), and two inverted repeat regions (IR_a_ and IR_b_; 25,140 bp each). The overall GC content was 37.6% and the plastome contained 130 genes, including 85 protein-coding, 37 tRNA and 8 rRNA genes.

The maximum likelihood (ML) analysis with 1,000 bootstrap replications was conducted using IQ-TREE v.1.6.7 (Nguyen et al. 2015). Seventeen representative species of Asteraceae (16 from tribe Cichorieae and one outgroup from tribe Astereae), including *I. repens*, were aligned using MAFFT v.7 (Katoh and Standley 2013). *Aster tataricus* (tribe Astereae) was used as an outgroup. The ML tree (Figure 1) showed that the genus *Ixeris* is a monophyletic and *I. repens* is closely related to *I. polycephala*, only available congeneric species with the complete plastome sequence. Based on currently all available plastome sequences of the five major clades within the subtribe Crepidinae (a total of five genera), the ML tree suggested that the subtribe Crepidinae is monophyletic (100% bootstrap support, BS) and that Lactucinae is sister to the Crepidinae (96% BS). A sister relationship between Hyoseridinae and Hypochaeridinae, albeit very weakly (53% BS), was suggested (Figure 1). Within the Crepidinae, the *Crepidiastrum*-*Lapsanastrum*-*Youngia* clade recognized by Kilian et al. (2009) was strongly supported (100% BS), while the *Ixeris*-*Ixeridium*-*Taraxacum* clade was not supported. Rather, the complete plastome phylogeny suggested that *Taraxacum* is more closely related to the genera of Crepidinae than to genus *Ixeris*. The plastid phylogenomic study based on much broader species coverage among subtribes of Cichorieae as well as within subribe Crepidinae will shed light on overall intersubtribal relationships within and phylogenetic and biogeographic history of genus *Ixeris* and related genera, respectively.

**Figure 1.**
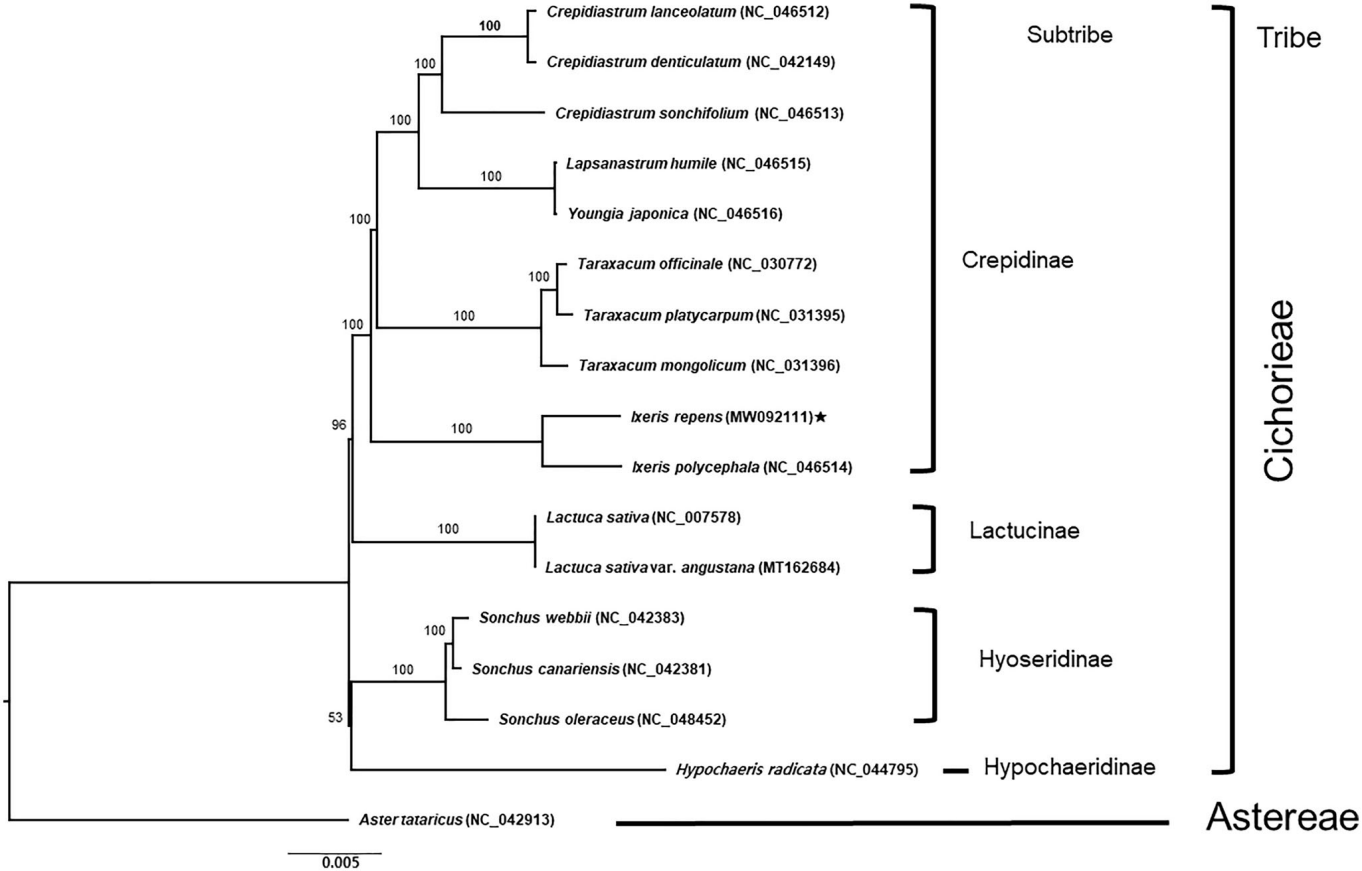
The maximum-likelihood (ML) tree based on 16 representatives of Asteraceae (four subtribes of Cichorieae) and one outgroup taxon, *Aster tataricus* (Asteraceae). The bootstrap support value based on 1,000 replicates is shown on each node.

## Data Availability

The genome sequence data that support the findings of this study are openly available in GenBank of NCBI at https://www.ncbi.nlm.nih.gov/ under the accession no. MW092111. The associated BioProject, SRA, and Bio-Sample numbers are PRJNA697908, SRR13590698, and SAMN17676084, respectively.
